# Propensity of IgA to self-aggregate via tailpiece cysteine-471 and treatment of IgA nephropathy using cysteamine

**DOI:** 10.1172/jci.insight.150551

**Published:** 2021-10-08

**Authors:** Xinfang Xie, Li Gao, Pan Liu, Jicheng Lv, Wan-Hong Lu, Hong Zhang, Jing Jin

**Affiliations:** 1 Department of Medicine/Nephrology and Hypertension, Feinberg Cardiovascular and Renal Research Institute, Northwestern University Feinberg School of Medicine, Chicago, Illinois, USA.; 2 Department of Nephrology and; 3 Department of Cardiology, The First Affiliated Hospital of Xi’an Jiaotong University, Xi’an, China.; 4 Renal Division, Department of Medicine, Peking University First Hospital, Beijing, China; Institute of Nephrology, Peking University, Beijing, China.

**Keywords:** Autoimmunity, Autoimmune diseases

## Abstract

IgA nephropathy is caused by deposition of circulatory IgA1 in the kidney. Hypogalactosylated IgA1 has the propensity to form poly-IgA aggregates that are prone to deposition. Herein, we purified poly-IgA from the plasma of patients with IgA nephropathy and showed that the complex is susceptible to reducing conditions, suggesting intermolecular disulfide connections between IgA units. We sought to find the cysteine residue(s) that form intermolecular disulfide. Naturally assembled dimeric IgA, also known as secretory IgA, involves a J chain subunit connected with 2 IgA1 molecules via their penultimate cysteine-471 residue on a “tailpiece” segment of IgA heavy chain. It is plausible that, with the absence of J chain, the cysteine residue of mono-IgA1 might aberrantly form a disulfide bond in poly-IgA formation. Mutagenesis confirmed that cysteine-471 is capable of promoting IgA aggregation. These discoveries prompted us to test thiol-based drugs for stabilizing cysteine. Specifically, the cystine-reducing drug cysteamine used for treatment of cystinosis showed a remarkable potency in preventing self-aggregation of IgA. When administrated to rat and mouse models of IgA nephropathy, cysteamine significantly reduced glomerular IgA deposition. Collectively, our results reveal a potentially novel molecular mechanism for aberrant formation of IgA aggregates, to which the repurposed cystinosis drug cysteamine was efficacious in preventing renal IgA deposition.

## Graphical abstract

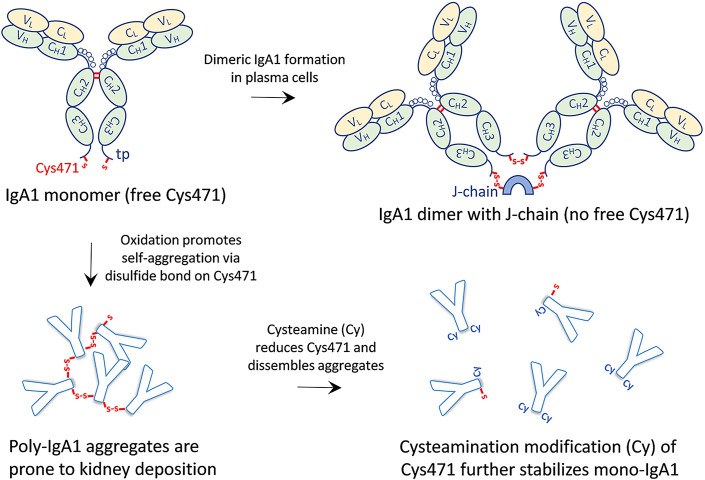

## Introduction

IgA nephropathy (IgAN) is the most common form of primary glomerulonephritis and a leading cause of end-stage kidney disease (ESKD) (1). The disease is characterized by chronic deposition of poly-IgA complexes in the glomerular mesangium, which causes inflammatory injuries to the kidney. Despite the prevalence of IgAN, the molecular mechanisms underlying IgA deposition in the glomerulus remain elusive, and specific treatments of the disease are currently unavailable.

There is an intense focus on characterizing the molecular features of poly-IgA, with regard to its propensity for renal deposition. It has been noted that high-molecular-weight poly-IgA complexes contain aberrant O-linked glycosylation of the hinge region of IgA1 heavy chain (2). In addition, glycoforms with reduced galactose contents are associated with higher incidence of IgAN (2–4). In-solution X-ray scattering data have shown that IgA1 is prone to nonspecific self-association when levels of O-galactosylation are reduced (5), in keeping with the notion that O-glycans protect IgA1 from self-aggregation and/or adhesion to matrices (6). It was also suggested that these aberrant glycoforms, referred to as galactose-deficient IgA1 (Gd-IgA1), are antigenic. In patients with IgAN, anti–Gd-IgA1 autoantibodies against IgG or IgM can be detected, and these IgG-IgA1 and IgM-IgA1 antibody-antigen pairs may lead to the formation of poly-IgA immune complexes in circulation that are susceptible to renal deposition (7).

Beside glycosylation on IgA1 hinge segment, another important feature of IgA molecule is that both IgA1 and IgA2 isotypes have secretory and nonsecretory forms. Secretory IgA (sIgA) has 2 IgA monomers linked by 2 additional polypeptide subunits, namely the J chain and the secretory component (SC). It is important to note that J chain and SC form cysteine-to-cysteine disulfide bridges with IgA heavy chain in the sIgA configuration. Specifically, J chain’s cysteine-14 and cysteine-68 residues form 2 separate disulfide bonds with cysteine-471 in the so-called secretory tailpiece of the IgA heavy chain to bridge 2 IgA molecules (8, 9). SC, on the other hand, forms a disulfide bond with cysteine-311 in CH2 domain of the IgA heavy chain (10). In the nonsecretory IgA1 monomer, which predominates in plasma, cysteine-311 and cysteine-471 residues are not connected to SC and J chain and, therefore, maintain their reduced and free forms. Under oxidative conditions, these free cysteines are prone to form disulfide bonds with other cysteine residues (11) that potentially coalesce IgA into high-order molecular aggregates. Our study investigated the propensity of free cysteine-311 and cysteine-471 of human IgA1 to promote self-aggregation as well as therapeutic means to disaggregate IgA complexes using thiol-reactive drugs.

## Results

### High-molecular-weight IgA complexes extracted from plasma from patients with IgAN contain intermolecular disulfide connections.

**Figure 1:**
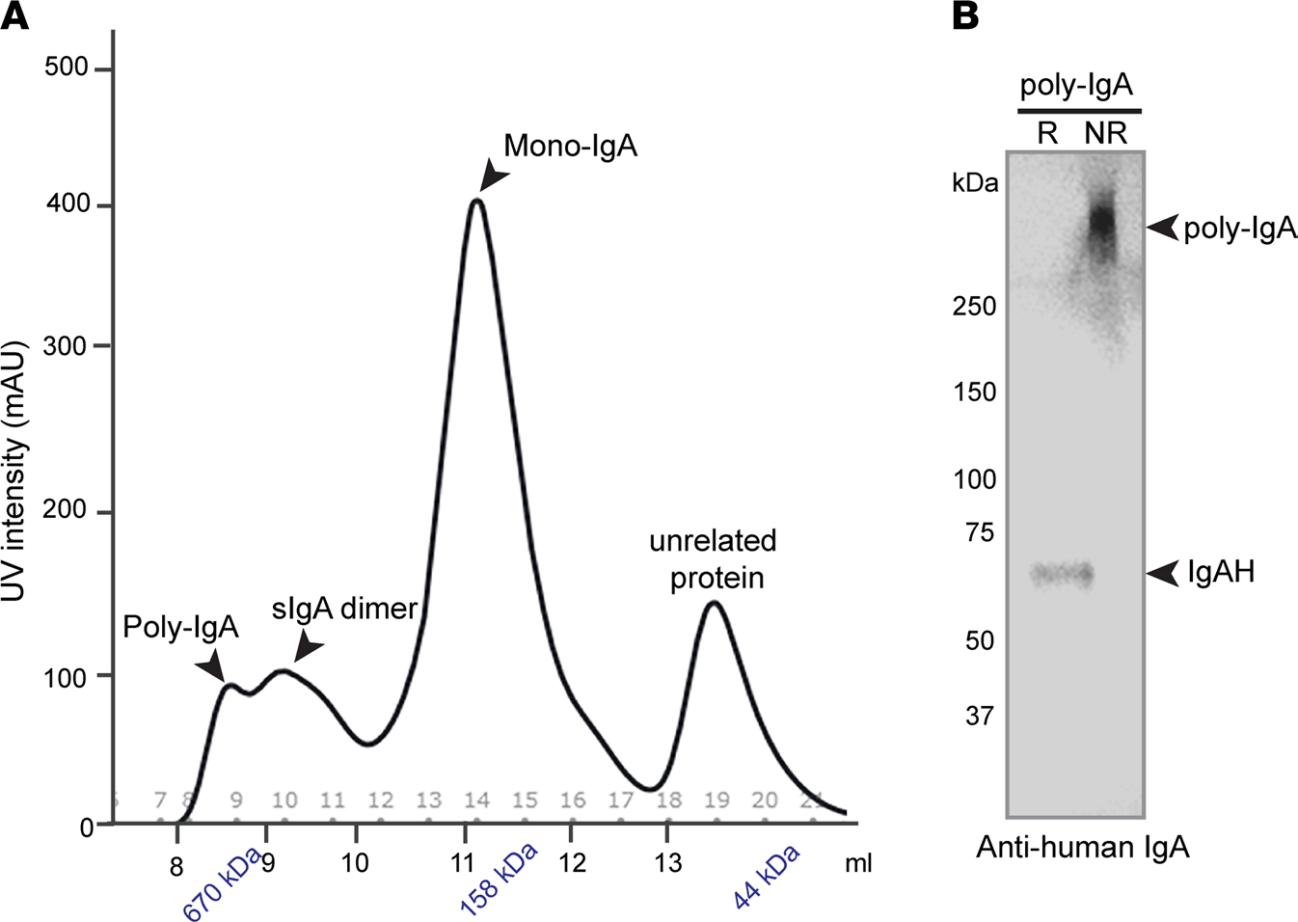
Plasma from patients with IgAN contains poly-IgA complexes that are susceptible to reducing agent TCEP. (**A**) Pooled plasma samples were collected from patients with IgAN (*n* = 8), and total IgA1 was extracted using jacalin-conjugated column. The extraction was then resolved by SEC, which was calibrated against molecular weight standards (arrowheads indicate kDa vs. elution volume along *x* axis). The IgA contents formed one major peak of mono-IgA, preceded by several overlapping minor peaks of poly-IgA and dimeric sIgA. (**B**) The poly-IgA fraction was subsequently analyzed by Western blotting with anti-human IgA heavy chain antibody under either reducing (R, with TCEP) or nonreducing (NR, without TCEP) conditions. With the addition of TCEP, the approximately 600 kDa poly-IgA complexes were reduced to an approximately 65 KDa band of IgA heavy chain (IgAH).

We sought to isolate high-molecular-weight IgA complexes from plasma of 8 patients with IgAN (clinical information is presented in Supplemental Table 1; supplemental material available online with this article; https://doi.org/10.1172/jci.insight.150551DS1). We followed a standard workflow to purify total IgA1 from pooled plasma using a jacalin-conjugated affinity column (12) (see Methods) and then subjected the extraction to size-exclusion chromatography (SEC). IgA1 monomers formed the dominant peak of approximately 160 kDa, preceded by dimeric sIgA of approximately 350 kDa (Figure 1A). Furthermore, poly-IgA1 formed additional overlapping minor peaks at >670 kDa (Figure 1A). By running SDS-PAGE of the poly-IgA fraction under both reducing and nonreducing conditions, it was determined that the molecular complexes of IgA1 were connected through disulfide bridges (Figure 1B). To be certain of the broad presence of reduction-sensitive IgA complexes, we extracted IgA1 complexes from individual patients by SEC and analyzed the contents by SDS-PAGE. These samples showed varying levels of high-molecular-weight IgA1 contents that could be dissociated by reducing agents (Supplemental Figure 1).

### Mutagenesis of rIgA mimetics identified penultimate residue cysteine-471 in promoting IgA aggregation.

**Figure 2:**
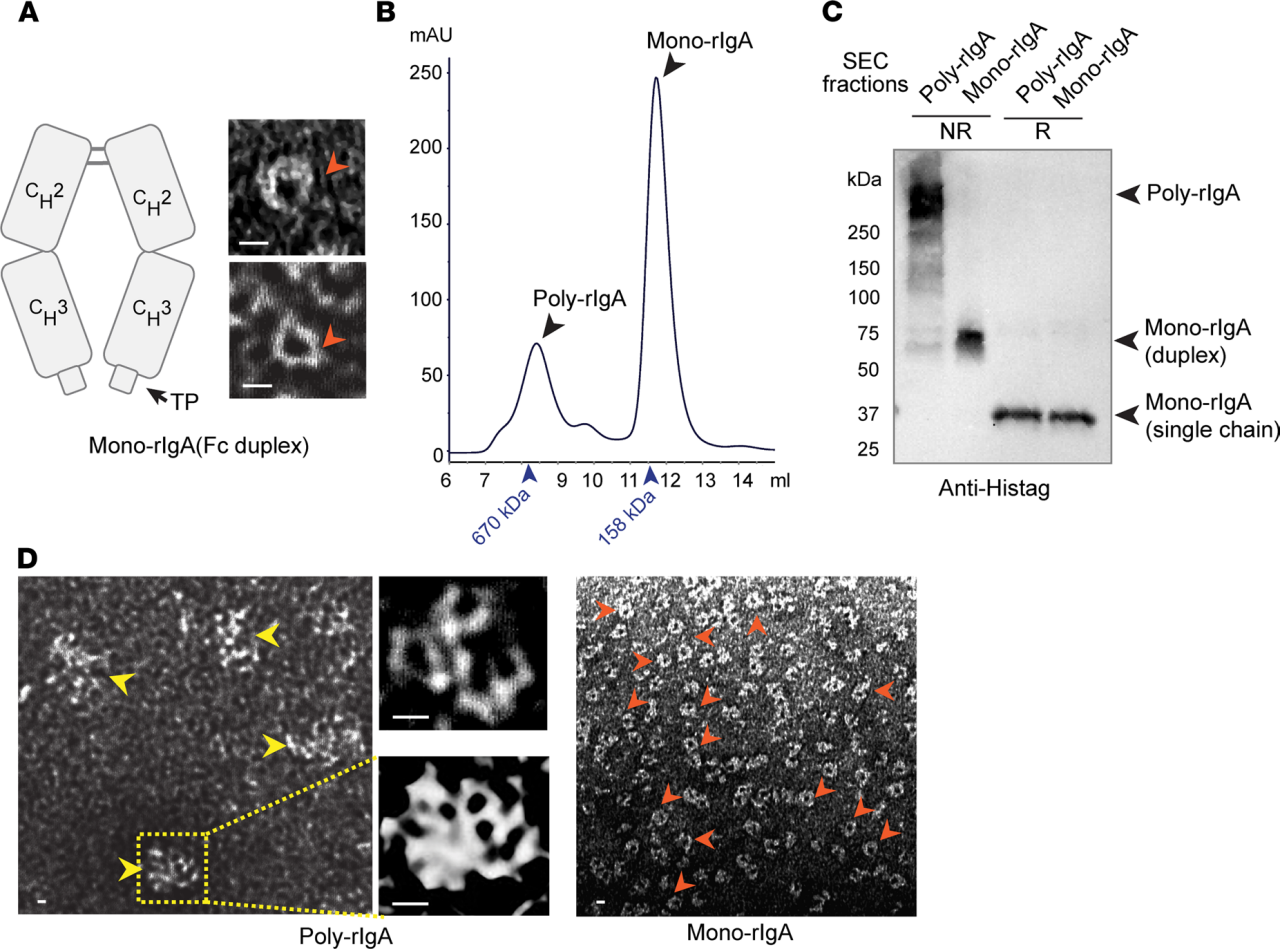
Intermolecular disulfide bond(s) involved in the self-aggregation of recombinant poly-rIgA. (**A**) Recombinant human and rat IgA mimetics comprising the CH2-CH3-Tailpiece (TP) segment of IgA heavy chain. Similar to native IgA heavy chain, the mimetics form a duplex that is referred to as mono-rIgA. TEM images confirmed rat rIgA duplexes in donut-like appearances. (**B**) Rat rIgA was resolved by SEC with a clear separation of its poly- and mono-rIgA contents. (**C**) SDS-PAGE results confirmed the presence of disulfide connections among self-associated rIgA in poly-rIgA complexes (NR, nonreducing condition): Under reducing conditions (R), both poly- and mono-rIgA reduced to single chains of 32 kDa. (**D**) TEM images of poly-rIgA SEC fraction showed rIgA aggregates (left, yellow arrowheads). High-magnification images (middle) show structures with multiple circular voids of monomeric rIgA units, in contrast to mono-IgA, which appeared as single donut-like structures (red arrowheads). Scale bar: 10 nm. Other examples of TEM images are shown in Supplemental Figure 2.

**Figure 3:**
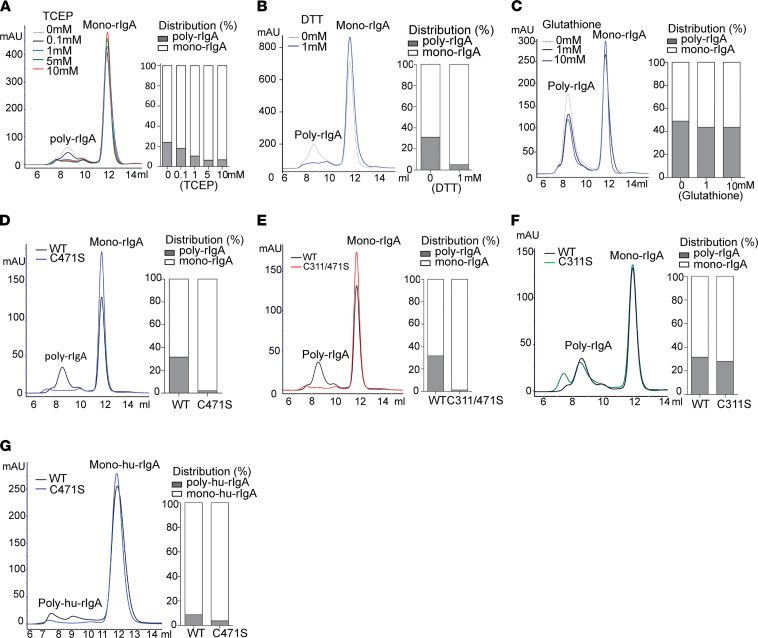
Mutagenesis analyses of cysteine-311 and cysteine-471 regarding formation of intermolecular disulfide bond(s). (**A–C**) SEC analyses of rIgA in the presence of TCEP, DTT, or glutathione, respectively. (**D–F**) SEC tracing of C471S, C311/471S, and C311 mutations of rIgA as compared with wild-type, respectively. The bar graphs show quantitation of poly- versus mono-rIgA contents based on AUC. (**G**) Comparing HEK293-produced human rIgA (hu-rIgA) wild-type and C471S mutant in terms of their poly-hu-rIgA contents as revealed by SEC.

To further investigate specific cysteine residue(s) involved in IgA complexes, we constructed expression vectors to produce recombinant IgA (rIgA) Fc segments of rat and human sequences (Figure 2A). Similar to native IgA heavy chain, these single-chain rIgAs also folded into duplexes, which we referred to as mono-rIgA, in keeping with tradition. We produced rat rIgA from bacterial expression (see Methods). Rat rIgA was resolved by SEC (Figure 2B), with a major peak of mono-rIgA duplex at approximately 64 kDa, preceded by a minor peak of poly-rIgA at >500 kDa. SDS-PAGE results further confirmed intermolecular disulfides in connecting rIgA units (Figure 2C). Transmission electron microscopy (TEM) showed interconnected rIgA structures in high-order complexes (Figure 2D and Supplemental Figure 2). To further demonstrate that poly-rIgA was also linked by disulfide bridges, we added reducing agents, such as Tris(2-carboxyethyl)phosphine hydrochloride (TCEP), 1,4-dithiothreitol (DTT), or glutathione, to rIgA. SEC analyses showed poly-rIgA disassembled into monomers by TCEP and DDT and, to a lesser degree, by glutathione (Figure 3, A–C). Specifically, SEC analyses showed a concentration-dependent reduction of the high-molecular-weight poly-rIgA peak by TCEP. Meanwhile, there was a slight compensatory increase of the mono-rIgA content (Figure 3A), as expected.

Considering that our fragment contained cysteine-311 and cysteine-471, which, in the absence of J chain and SC, were in their free forms and available for connecting other rIgA units, we sought to conduct mutagenesis studies of these 2 cysteines. cysteine-311 and cysteine-471 were either individually, or together, mutated to serine (S). These C311S and C471S single mutants and C311/471S double mutant were produced as rat rIgA proteins. By examining the SEC traces of these mutants, we noticed that, while the monomers of all variants appeared the same, there were dramatic differences in the poly-rIgA contents. Notably, the prominent high-molecular-weight peak for poly-rIgA completely disappeared in the C471S single mutant and C331/471S double mutant (Figure 3, D and E). Meanwhile, C311S alone still had poly-rIgA contents, albeit eluted at a different time in SEC than the wild-type protein (Figure 3F). In addition, SDS-PAGE results further confirmed cysteine-471’s involvement in poly-rIgA formation (Supplemental Figure 3A).

In parallel, we repeated the experiments using human rIgA1-Fc and its C471S mutant, which were produced from mammalian cell expression (see Methods). Wild-type rIgA1 also had both monomer and polymer contents, in contrast to C471S, which showed a greatly reduced level of poly-rIgA1 (Figure 3G and Supplemental Figure 3B), suggesting that cysteine-471 promotes rIgA self-association.

### Cysteamine stabilizes cysteine-471 and reduces poly-IgA levels.

**Figure 4:**
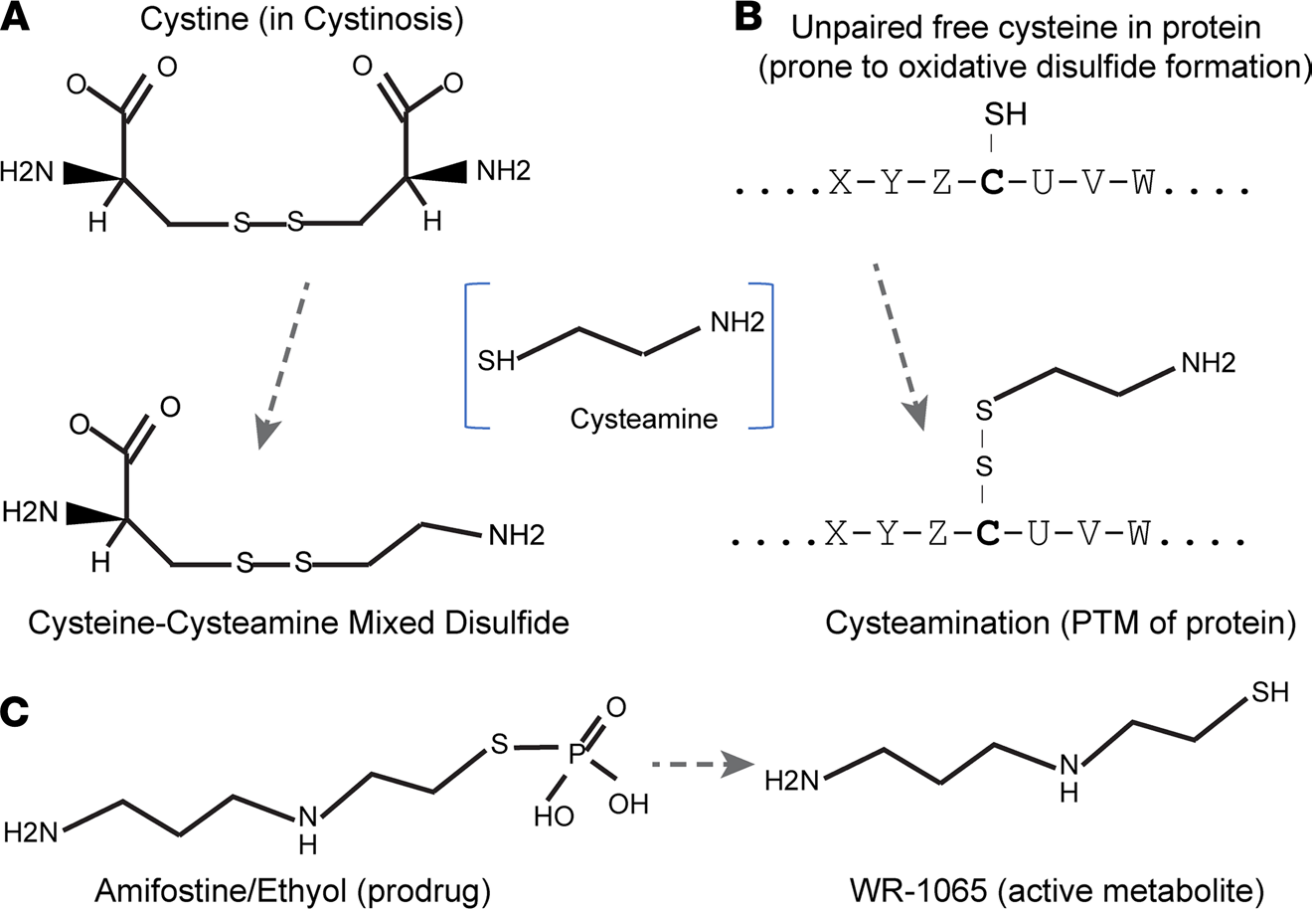
Cysteamine reacts with amino acid cysteine or free cysteine in protein. (**A**) Drug action of cysteamine in treatment of cystine crystals in cystinosis via formation of cysteine-cysteamine (soluble) mixed disulfide. (**B**) Both human cell-produced endogenous cysteamine and cysteamine drug interact with susceptible cysteine residues in proteins, such as cysteine-471 in human IgA1, as a form of posttranslational modification (PTM). (**C**) Another aminothiol drug amifostine (Ethyol) is a prodrug; its active metabolite WR-1065 is capable of interacting with small thiol molecules as well as unpaired cysteine residues in proteins.

**Figure 5:**
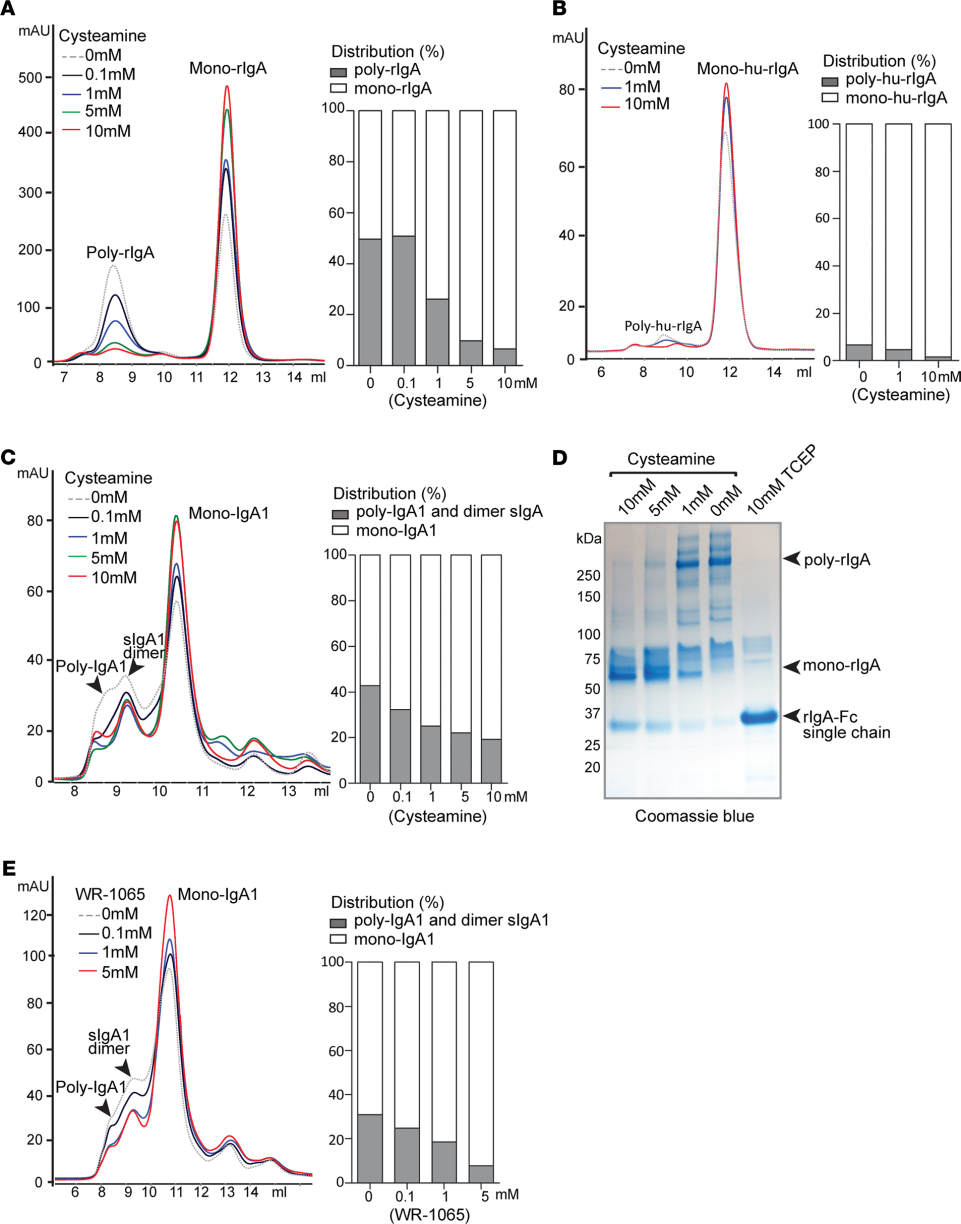
Cysteamine treatment of either recombinant rIgA or native human IgA ex vivo lowers poly-IgA contents. (**A**) Cysteamine treatment of rat rIgA (produced by *E*. *coli*) showed greatly reduced poly-rIgA peak in SEC tracing. (**B**) Human recombinant rIgA (hu-rIgA) produced by HEK293 cells has a lower level of poly-rIgA as compared with that shown in **A**. Treatment with cysteamine lowered the poly-rIgA peak in SEC tracing. (**C**) Using total IgA1 extracted from patients with IgAN, ex vivo treatment with cysteamine also lowered poly-IgA1 levels. (**D**) SDS PAGE analyses showed dose-dependent responses of poly-rIgA reduction by cysteamine. The reactions produced duplex mono-rIgA with 2 rIgA chains that remained connected. In contrast, TCEP completely separated paired rIgA heavy chains and resulted in single chain rIgA-Fc. (**E**) Aminothiol WR-1065 also lowered poly-IgA1 contents as shown by SEC.

After determining that the tailpiece cysteine connects IgA molecules that form aggregates, we sought to test the ability of interventional drugs to disassemble IgA complexes by reducing the disulfide bond on cysteine-471. Cysteamine, an aminothiol that can react with cysteine, is both a natural metabolite produced in mammalian cells and a clinical drug used for treatment of cystinosis (13). In cystinosis, cysteamine reduces the disulfide bond in cystine, which is the oxidated dimer of amino acid cysteine (Figure 4A). In the context of cysteine residues in proteins, both endogenous cysteamine and cysteamine administered as therapeutic drug can form disulfides with susceptible cysteine sulfhydryl groups in a process called cysteamination (14) (Figure 4B). We sought to determine whether cysteamine could reduce cysteine-471 disulfides to disaggregate poly-IgA. Rat rIgA was treated with cysteamine, and the protein complexes were analyzed by SEC. As expected, the drug effectively lowered poly-rIgA levels in a dose-dependent manner, whereas the relative amounts of mono-rIgA were slightly increased (Figure 5A). These results suggested that cysteamine was able to disassemble high-molecular-weight IgA complexes by disrupting intermolecular disulfide bond.

Human rIgA1 produced from mammalian expression had a smaller fraction of poly-rIgA contents as compared with *E*. *coli*–produced rat rIgA (compare Figure 3G and Figure 2B). Nevertheless, treatment of human rIgA with cysteamine also showed a reduction of poly-rIgA levels (Figure 5B). In addition, we purified human IgA1 whole molecules from pooled plasma of patients with IgAN and subjected the samples to different concentrations of cysteamine (Figure 5C). In a dose-dependent manner, cysteamine reduced the amounts of poly-IgA1, whereas the mono-IgA1 levels slightly increased in response to cysteamine, as expected. Because the dimeric sIgA1 peak partially overlapped with that of poly-IgA1 on SEC, it is difficult to accurately assess the impact of treatment to sIgA1 dimers. However, as the overall shape of the sIgA peak remained largely unchanged, we expected the contents of sIgA1 to remain stable. Collectively, these results indicated the structural susceptibility of cysteine-471 to cysteamine in poly-IgA1, in contrast to the stability of monomers.

To further ascertain the mechanism of action of cysteamine in targeting disulfides, we performed nonreducing SDS-PAGE analysis of rat poly-rIgA (Figure 5D). Samples were pretreated with 0–10 mM cysteamine, or 10 mM TCEP as positive control. As expected, poly-rIgA appeared as >250 kDa bands in SDS-PAGE. A dose-dependent response of poly-rIgA to cysteamine was evident, with the majority of the protein running at approximately 64 kDa in SDS-PAGE, consistent with disaggregated monomers. Meanwhile, treatment with 10 mM of TCEP resulted in further reduction of the molecular weight to approximately 32 kDa as a single-chain protein, indicating normal disulfide bridges between IgA heavy chains were being disrupted. These results suggested that modest reduction activity of cysteamine, as compared with TCEP, was effective in reducing vulnerable intermolecular disulfides between poly-IgA units, while leaving normal disulfides between paired IgA heavy chains intact.

### WR-1065, the active metabolite of radioprotective drug amifostine, reduces poly-IgA levels ex vivo.

Another thiol-based drug, amifostine (also known as ethiofos; Ethyol) (Figure 4C), is a clinical radioprotector and cytotoxic chemoprotector (15). Its active metabolite WR-1065 is an aminothiol that, similar to cysteamine, can react with cysteine as well as detoxify nonprotein metabolites (16). We added WR-1065 to reactions with total IgA1 extracted from human sera. As expected, SEC analyses showed a reduction of poly-IgA levels following treatment (Figure 5E).

### In vivo treatment with cysteamine reduces glomerular mesangial IgA deposition in murine models.

**Figure 6:**
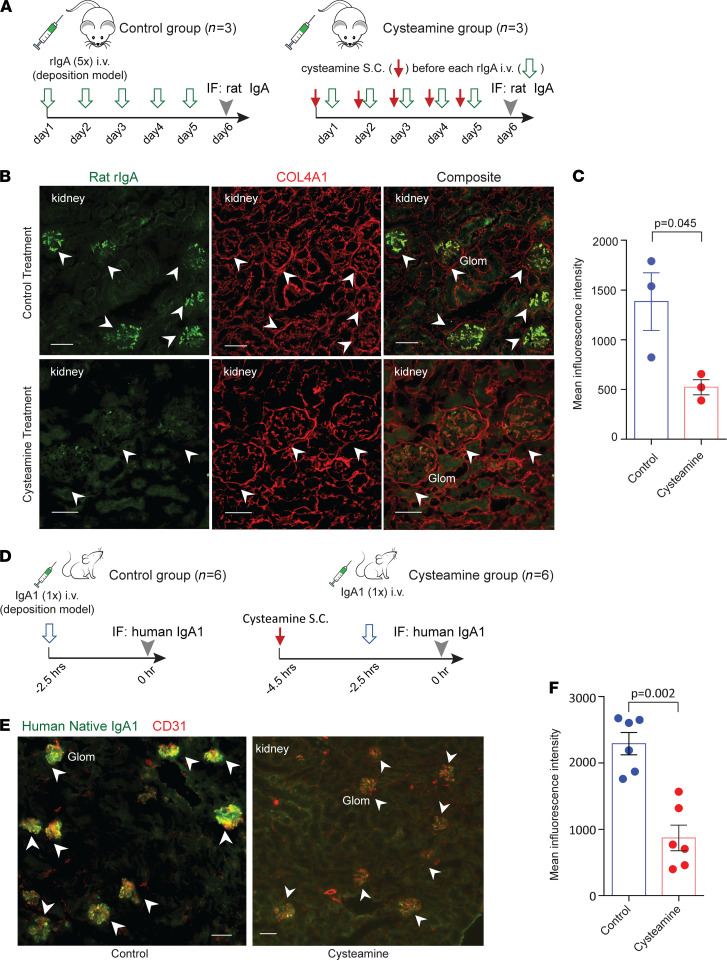
In vivo treatment of rats and mice with cysteamine lowers IgA deposition in the kidney in injection-induced IgAN models. (**A–C**) Rat model treated with cysteamine. (**A**) In a rat IgA deposition model, rats received a daily dose of cysteamine, or buffer control, followed by an injection of recombinant rat rIgA for 5 consecutive days. (**B**) Representative immunofluorescence images showed prominent rIgA deposition in glomeruli (Glom; arrowheads) in rats treated with buffer (*n* = 3), in contrast to weaker deposits in cysteamine-treated rats (*n* = 3). Additional kidney images are provided in Supplemental Figure 4A. (**C**) Quantitation of deposit in glomeruli between control and cysteamine treatment groups compared by *t* test (mean ± SEM, 1384 ± 290 MFI vs. 523 ± 76 MFI, *n* = 3 in each group). Significance between the 2 groups was determined by 2-tailed *t* test. (**D**) Mouse model of IgA deposition from injection of human IgA1 purified from human plasma. Each mouse was injected with a single dose of purified human IgA1 2 hours after pretreatment with either cysteamine or buffer control. (**E**) The buffer control mouse group (*n* = 6) had prominent IgA1 deposition in glomeruli (arrowheads). In contrast, pretreatment of the mice with cysteamine (*n* = 6) greatly reduced IgA1 deposition. Additional immunofluorescence images are shown in Supplemental Figure 4B. (**F**) Quantification of glomerular IgA1 intensity between buffer and cysteamine treatment groups (mean ± SEM, 2293 ± 163 MFI vs. 870 ± 193 MFI, *n* = 6 in each group). Significance between the 2 groups was determined by 2-tailed *t* test. Scale bar: 50 μm.

We sought to test the in vivo efficacy of cysteamine in a rat model of IgA deposition. We used a passive induction model with 5 daily i.v. injections of purified recombinant rat rIgA in rats (17) (Figure 6A, left). Rats developed prominent IgA deposition in the glomerulus (Figure 6B, top). Meanwhile, rats that also received subcutaneous doses of cysteamine 2 hours prior to each rIgA injection (Figure 6A, right) showed less glomerular deposition (Figure 6B, bottom; Figure 6C; and Supplemental Figure 4A). In order to confirm that rIgA deposition was attributable to disulfide-connected poly-IgA, and reduction of its signals in the glomerulus by cysteamine treatment was due to disassociation of the complexes, we separately purified mono-rIgA and poly-rIgA by SEC. As expected, injection of the poly-rIgA fraction resulted in mesangial deposition signals, whereas injection of the mono-rIgA fraction did not cause glomerular deposition (Supplemental Figure 5).

Next, we sought to examine the treatment response of human IgA to cysteamine. We used another passive induction model for IgA deposition with injections of human IgA1 in mice (18). Mice, unlike humans and rats, do not have IgA Fc receptor CD89/FcαRI, which can eliminate poly-IgA complexes by phagocytic cells, including macrophages, neutrophils, and Kupffer cells (19). We performed a 1-time i.v. injection in mice with total IgA1 purified from human plasma (see Methods) (Figure 6D, left). As expected, immunofluorescence staining of kidney specimens showed glomerular deposition of human IgA1, prominently located along capillary loops and in mesangial areas (Figure 6E, left). In contrast, pretreatment of mice with a subcutaneous dose of cysteamine at 200 mg/kg BW 2 hours prior to IgA injection (Figure 6D, right) showed markedly lower glomerular IgA signals (Figure 6E, right). Particularly, the broad presence of high intensity IgA1 puncta mostly disappeared in the animals that received this prophylactic dose of cysteamine (Figure 6F and Supplemental Figure 4B). Meanwhile, blood IgA1 levels showed no significant difference between the 2 groups of mice (Supplemental Figure 4C), demonstrating specific reduction of IgA deposition in the kidney by cysteamine.

## Discussion

Our study investigated structural features of IgA that could potentially make it susceptible to forming high-order complexes and causing IgA nephropathy. Unexpectedly, we discovered poly-IgA complexes isolated from patient plasma disassembled by reducing agents, suggesting that IgA and possibly also its non-IgA constituents interconnected via disulfide bridges. In addition to the cysteine residues that participate in disulfide formation between the pair of IgA heavy chains and between heavy and light chains, there are 2 additional cysteines, cysteine-311 and cysteine-471, involved in linking SC and J chain subunits of dimeric IgA (20), respectively. However, because mono-IgA1 is the main circulatory form in blood, these 2 cysteine residues are expected to be in their free and reduced forms and, therefore, susceptible to oxidation. We further discovered that cysteine-471 in particular can mediate aberrant self-association of IgA and promote glomerular deposition of the resulting poly-IgA complexes. We then sought the use of aminothiol drugs, such as cysteamine and WR-1065, to stabilize mono-IgA and prevent poly-IgA–directed kidney deposition. Ex vivo and in vivo results showed that these interventional drugs were efficacious in preventing IgA self-association and its glomerular deposition in rodent models.

Admittedly, it remains unclear as to whether the mechanism of action with cysteamine was through disassembling of poly-IgA1 in circulation, in renal deposits followed by accelerated clearance, or both. We should also note that cysteamine is not a potent reducing agent that can effectively break existing disulfide bonds (Figure 5D). Instead, therapeutic cysteamine possibly protects free cysteine-471 of IgA1 via cysteamination to lower its reactivity toward IgA1 or other plasma and matrix proteins. Generally, intermolecular disulfide reactions are stochastic, but this is facilitated by noncovalent interactions between two proteins to position a pair of cysteine residues in proximity, often at interaction interfaces (21). Therefore, it is conceivable that any normal disulfides that are integral parts of immunoglobulin folds, such as those between IgA heavy chains (as in Figure 5D), or between the heavy chain and the light chain (not tested) (5), could withstand cysteamine treatment. In other words, aberrantly connected disulfide interactions are expected to be more susceptible to the drugs. Nevertheless, alkylating agents that form irreversible bonds with cysteine residues, such as N-ethylmaleimide or iodoacetamide, risk destabilizing the entire IgA molecule and may not be desirable for treatment as compared with milder aminothiols such as cysteamine. With regard to the in vivo models in our study, we caution that they do not fully resemble the clinical development of IgA nephropathy, which typically follows a slow course of progression over a long period of time. This is likely due to chronic exposure of patients to low levels of injurious poly-IgA complexes. In contrast, the passive induction models with bolus doses of exogenous IgA presented spikes of poly-IgA levels in blood, causing acute deposition in the kidney. Because mild aminothiols function by shifting the dynamic equilibrium between free and disulfide cysteines, chronic models that fully phenocopy clinical IgAN are better choices for evaluating treatment effects.

Although we discuss the intrinsic propensity of IgA1 to self-aggregate via intermolecular disulfides, our findings do not contradict with the established “galactosylation-centric” theory of IgA nephropathy. Structural evidence suggests that hypogalactosylated IgA may be prone to self-aggregation (5, 6). In addition, in bioengineering of therapeutic IgA drugs, eliminating tailpiece improves IgA solubility (22). These studies suggest that noncovalent coalescence of hypogalactosylated IgA units could facilitate disulfide connectivity of IgA tailpiece in the first place, which, in turn, enhances self-association. Similarly, the well-accepted multihit model for the pathogenesis of IgA nephropathy includes antigenicity of Gd-IgA in inciting antiglycan autoantibodies in promoting IgA immune complexes (2, 23, 24). It is still plausible that these antibody-antigen interactions bring IgA monomers together for their reactive cysteine-471 to form disulfide bridges with additional serum and/or matrix proteins.

Identifying the underlying biochemical processes has clinical implications toward discovering new therapies for IgAN, including with existing drugs. Both cysteamine and WR-1065/2-((aminopropyl)amino) ethanethiol have been extensively formulated for treating other conditions. Cysteamine bitartrate capsulated for delayed release (Procysbi and Cystagon) is an improved formulation for oral administration, whereas amifostine is a prodrug form for i.v. administration (Figure 4C). From a practical perspective, cysteamine bitartrate represents a more suitable option than amifostine, considering the requirements of long-term treatment for most patients with IgAN.

## Methods

### Recombinant construction of rat and human IgA mimetics.

The DNA sequence that encodes the wild-type rat IgA-Fc segment of CH2-CH3-TP was cloned into PET30a vector (Invitrogen) with an N-terminus 6×His-tag. Corresponding point mutations of C471S, C311S, and C311/471S were generated by site-directed mutagenesis. Human IgA1-Fc CH2-CH3-TP cDNA and its mutant for C471S were fused to sequences encoding IL-2 signal peptide and 6×His-tag at the 5′-end in pcDNA3 vector (Invitrogen). Methods for protein expression and purification were described previously (17). Briefly, rat IgA analogs were expressed in the BL21 DE3 strain of *E*. *coli*, and recombinant protein were purified using Histrap HP columns (GE Healthcare), whereas human rIgA mimetics were produced from human embryo kidney (HEK293) cells by transfection of the plasmids. Description of native IgA purification from plasma is in the Supplemental Methods.

### Analysis of intermolecular disulfide bond formation in IgA self-aggregates.

The overall complex size of IgA aggregates was determined either by SEC or SDS-PAGE. For evaluating the involvement of intermolecular disulfide connectivity in IgA complexes, IgA samples were treated with either reducing agents, such as DTT, TCEP, or reduced glutathione (MilliporeSigma), or interventional drugs, such as cysteamine (MilliporeSigma) and WR-1065 (MilliporeSigma). Before running on SEC, samples were treated with the drugs at indicated concentrations at 37°C for 1 hour.

### Cysteamine treatment of IgA deposition in rat and mouse model.

To establish the passive rat model, 5 mg/kg BW recombinant rat rIgA, which contained a fraction of poly-rIgA, was i.v. injected daily to six 14-week-old male Wistar rats (Charles River Labs) for 5 consecutive days. Every day, 3 rats in each group received a subcutaneous dose of either 250 mg/kg cysteamine or buffer control 2 hours before the rIgA injection. Twenty-four hours after the last injection of rIgA, kidneys were collected for immunofluorescence detection of deposits with goat anti-rat IgA antibody (catalog STAR111, Bio-Rad). Similarly, a passive IgAN mouse model was established by injecting 35 mg/kg purified human IgA1 in BALB/c mice (Charles River Labs). Two hours before IgA1 injection, 6 mice in each experimental group each received a pretreatment dose of either 200 mg/kg cysteamine or PBS via subcutaneous injection. Two and a half hours after human IgA1 injection, kidneys were harvested and specimens were stained with FITC-conjugated anti-human IgA antibody (catalog 2050-02, SouthernBiotech). Plasma samples were collected at 0.5 hours, 1 hour, and 2 hours after IgA1 injection of all mice. Injected human IgA1 concentrations in mouse serum were detected by ELISA with anti-human IgAα chain antibody (catalog 2052-01, SouthernBiotech) coated and captured by anti-human Ig Fab-HRP antibody (catalog 2010-05, SouthernBiotech). Methods of immunofluorescence and quantification are described in the Supplemental Methods.

### Statistics.

Data are displayed graphically, and statistical analyses were performed using GraphPad Prism 5.0. Group data are reported as mean ± SEM. Significance between 2 groups was determined by 2-tailed *t* test. *P* values of less than or equal to 0.05 were considered significant.

### Study approval.

All animal studies were carried out in accordance with NIH regulations for the care and use of laboratory animals and following Northwestern University IACUC–approved protocols (IS00009990, OLAW: A3283-01). Patient samples were collected following approval by the ethics committee of Peking University First Hospital in accordance with the principles of the Declaration of Helsinki. All patients who donated plasma samples provided informed consent in writing before inclusion in this study.

## Author contributions

XX and JJ conceived of the presented idea. XX developed the study and performed the experiments. LG and PL assisted with the experiments. XX and JJ performed the analyses, drafted the manuscript, and assembled the supplemental figures. WL, JL, and HZ revised the manuscript. All authors discussed the results and commented on the manuscript. 

## Supplementary Material

Supplemental data

## Version 1 08/24/2021

In-Press Preview

## Version 2 10/08/2021

Electronic publication
