# Supplementary material for: Propensity of IgA to self-aggregate via tailpiece cysteine-471 and treatment of IgA nephropathy using cysteamine
Source: JCI Insight. 2021 Oct 8;6(19):e150551. doi: 10.1172/jci.insight.150551 (PMC8525636; doi:10.1172/jci.insight.150551)
Supplement: Supplemental data [file jciinsight-6-150551-s222.pdf]

Supplementary Information:

Propensity of Immunoglobulin A Self-aggregation via 'Tailpiece' Cysteine-471 and Treatment of IgA Nephropathy Using Cysteamine

By Xinfang Xie, Li Gao, Pan Liu, Jicheng Lv, Wanhong Lu, Hong Zhang and Jing Jin

Table of Contents

1. Materials and Methods ……………………………………………………………………………………………………..3
2. Supplementary Tables

   Supplementary Table S1. Baseline characteristics of IgA nephropathy for plasma collection……………4

   Supplementary Figures

   a. Supplementary Figure S1. Individual IgA nephropathy patients' poly-IgA complexes disassembled by reducing agent………5
   b. Supplementary Figure S2. Transmission electron microscopy of recombinant rat poly- and mono-rIgA………6
   c. Supplementary Figure S3. Tailpiece residue cysteine-471 of IgA mediates IgA aggregation via intermolecular disulfide ……………………………………………………………………………………………………7
   d. Supplementary Figure S4. *In vivo* treatment of rats and mice with cysteamine lowers IgA deposition in the kidney……………………………………………………………………………………………………….8
   e. Supplementary Figure S5. Purified recombinant poly-IgA fraction causes glomerular deposition in rats……….9

Supplementary Methods

IgA purification from plasma

IgAN patients who donated plasma samples had provided informed consent in writing before study inclusion and their diagnoses were based on kidney biopsy. IgA1 from IgAN patient sera was purified by Jacalin (Thermo Scientific, USA)-directed affinity chromatography. Poly-IgA1 contents were further enriched via running Superdex 200 Increase gel filtration molecular sieve (GE Biosciences) and collecting high-molecular weight fractions from an AKTA protein purification system (GE Biosciences).

SDS-PAGE and Western blotting

Proteins in sample buffer (Bio-Rad Laboratories, Hercules, CA, USA) with or without TCEP or 2-mercaptoethanol (for reducing or nonreducing condition, respectively) were resolved by 4−12% SDS-PAGE (Bio-Rad Laboratories). rIgA bands were subsequently visualized either by staining with GelCode Blue (Thermo Fisher Scientific), or by Western blotting on PVDF membrane. For Western blotting, 5% non-fat milk was used in blocking for one hour at room temperature. The membrane was then incubated with mouse anti-Histag antibody (Thermo Scientific), or HRP-conjugated goat anti-rat IgA α-chain antibody (Cat. ab97185, Abcam, UK), or goat anti-human IgA HRP antibody (Cat:2050-05, SouthernBiotech) or goat anti-human IgA1 HRP antibody (Cat:9130-05, SouthernBiotech) for detecting rat or human IgA (Fc) or human IgA1 (Fc), respectively. The membrane was developed using the Clarity™ ECL substrate (Bio-Rad Laboratories, CA, USA).

TEM analyses

Structural characterizations of purified mono-rIgA and poly-rIgA were performed with transmission electron microscopy (TEM) as described previously(1). TEM analyses of the structures of poly-rIgA and mono-rIgA were conducted following a standard negative staining protocol. In brief, purified poly-rIgA or mono-rIgA was diluted in PBS to a concentration of 100 μg/ml. A 10 μl droplet was applied to a glow-discharged carboncoated copper grid and allowed to sit for 1 min. The grid was washed by dipping in two separate drops

in water followed by two drops in 2% uranyl acetate (Electron Microscopy Sciences). Grids were examined at the Northwestern Electron Probe Instrumentation Center (EPIC) using Hitachi HT‐7700 Biological S/TEM Microscope.

Immunofluorescence staining

Animal studies were approved by Northwestern University IACUC committee (Protocol: IS00009990). Frozen tissues were sectioned at 4 µm for IgA detection using goat anti-rat IgA (Cat:STAR111, Bio-Rad Laboratories) at 1:100, or anti-human IgA antibody (Cat:2050-02, Bio-Rad Laboratories) at 1:80 dilution. Anti-collage IV α1 (Cat:NB120-6586, Novus) at 1:500 dilution, rat anti-mouse CD31 (Cat:553370, BD Biosciences) at 1:100 dilution, Anti-collage IV α1 (1:500 dilution from Novus, CO, USA, Cat.577222) and DAPI were used as counterstaining. Immunofluorescence images were captured by Nikon Ti2 Widefield microscope. The mean immunofluorescence intensity per glomerulus area was derived from 15 glomeruli per kidney section assisted by Image J software.

Supplementary references

1. Booth DS, Avila-Sakar A, and Cheng Y. Visualizing proteins and macromolecular complexes by negative stain EM: from grid preparation to image acquisition. *J Vis Exp.* 2011(58).

Supplementary Table S1. Baseline characteristics of IgA nephropathy for plasma collection.

IgAN pts	Gender	Age	UTP	SCr	Alb	Hb	M	E	S	T	C
No.1	2	43	0.51	65	41.1	118	1	0	1	0	1
No.2	2	30	1.65	70	33.2	121	1	1	1	0	1
No.3	2	35	0.6	66	40.1	104	1	1	1	0	0
No.4	1	40	2.21	105	36.8	147	1	1	1	0	0
No.5	2	38	0.59	50	32.5	123	1	0	1	0	0
No.6	1	44	0.54	137	30.3	135	1	0	0	0	0
No.7	1	65	0.52	123	43	157	1	0	1	0	1
No.8	1	58	1.41	109	35.2	123	1	0	0	1	0

UTP: Urinary total protein, SCr: serum creatinine, Alb: serum albumin, Hb: hemoglobin. The Oxford Classification of IgA nephropathy: mesangial hypercellularity (M), segmental sclerosis (S), and interstitial fibrosis/tubular atrophy (T) lesions, crescents (C).

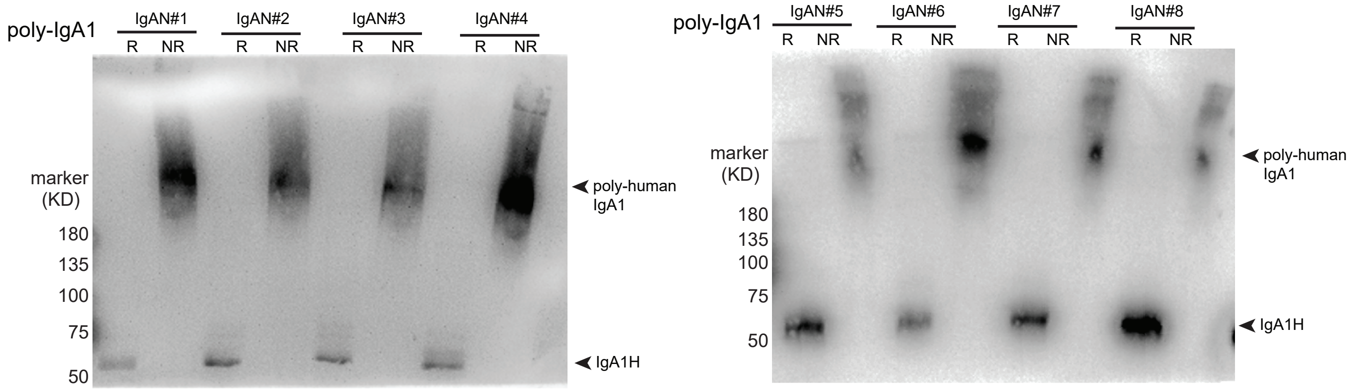

Supplementary Figure S1. Individual IgA nephropathy patients' poly-IgA complexes disassembled by reducing agent.
Plasma samples were collected from eight IgA nephropathy patients (#1-#8: clinical information in supplementary table S2). Following purification of total IgA1 by Jacalin beads, poly-IgA1 complexes were extracted by SEC. Purified complexes were treated in the presence or absence of 2-mercaptoethanol and were then resolved by SDS PAGE and anti-IgA Western blotting. In all samples, with the absence of 2-mercaptoethanol (NR), poly-IgA1 appeared at ~600 kDa. With 2-mercaptoethanol (R), a single ~65 kDa IgA1 band corresponding to heavy chain (IgA1H) was observed for each sample.

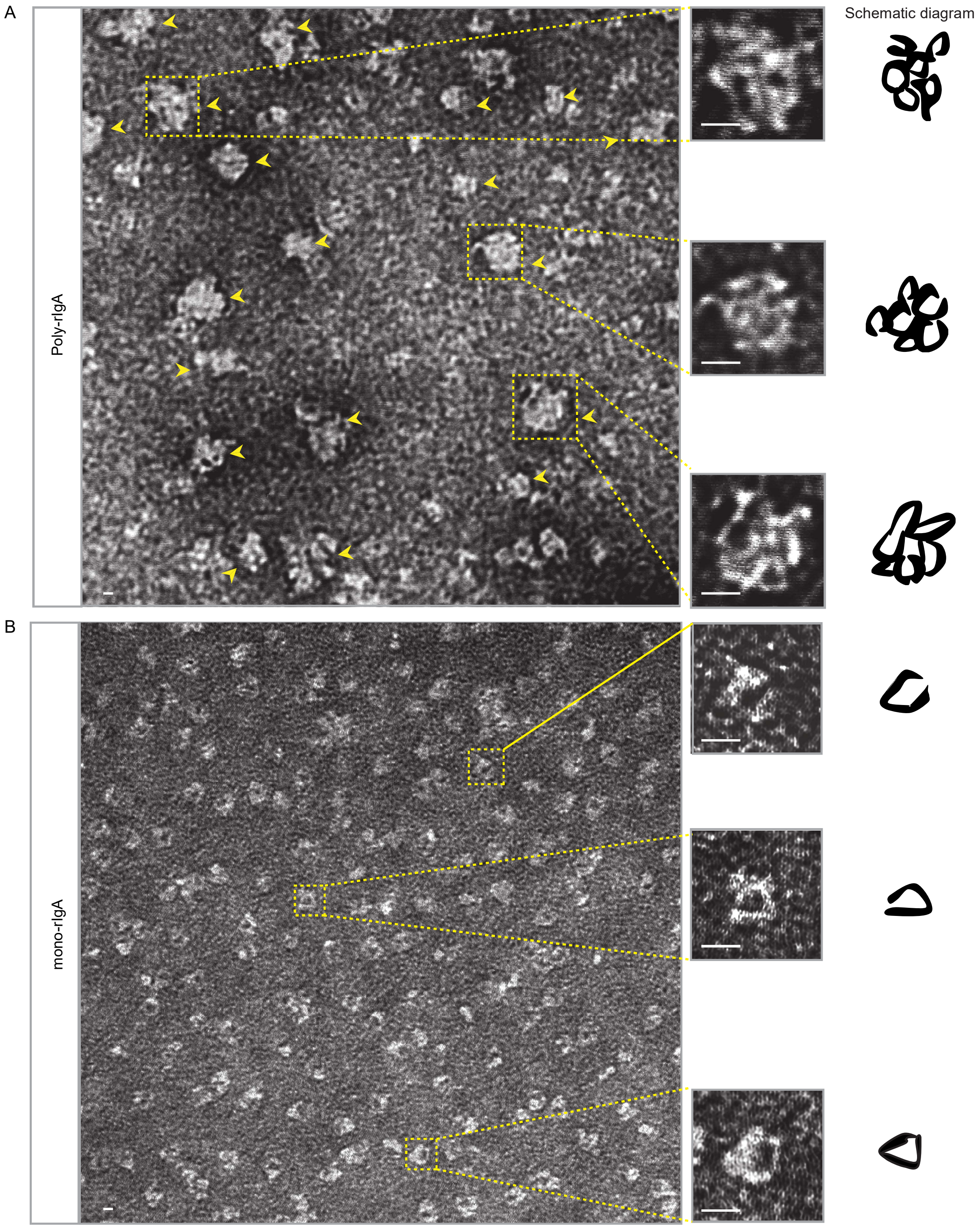

Supplementary Figure S2. Transmission electron microscopy of recombinant rat poly- and mono-rIgA.

The images were from the experiment described in Fig2D with separate collections of poly- and mono-rIgA fractions by SEC. **(A)**. This TEM image of poly-rIgA shows clusters of poly-rIgA complexes (left: yellow arrowheads; insets: complexes comprised of multiple donut-like rIgA units). Schematics are shown next on the right. **(B)**. Mono-rIgA appeared more uniformly as single donut-like structures (insets: selected single rIgA units with schematic diagrams next on the right). Scale bar: 10 nm.

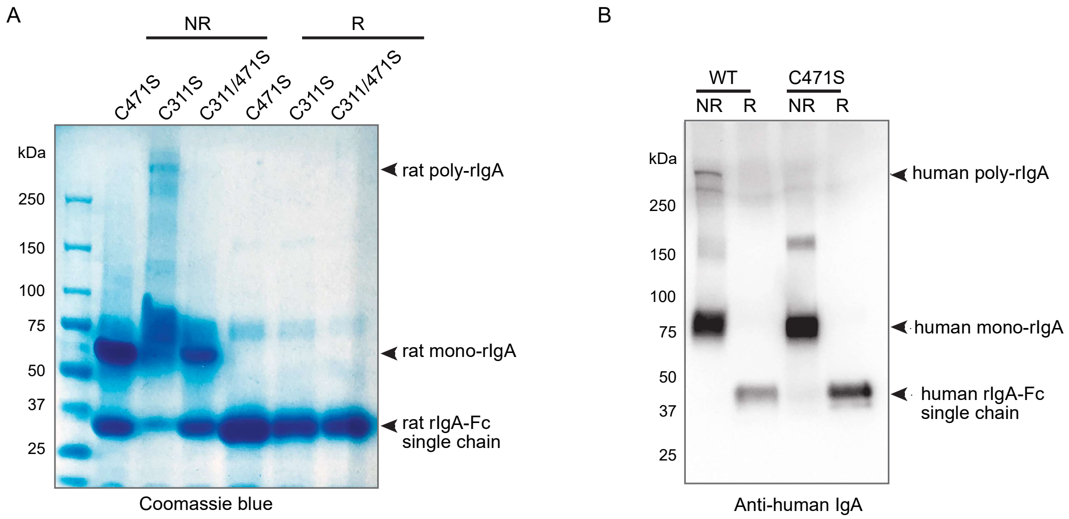

Supplementary Figure S3. Tailpiece residue cysteine-471 of IgA mediates IgA aggregation via intermolecular disulfide.

(A). SDS PAGE showed that Cys471-to-Ser substitution in C471S and C311/471S, but not Cys311-to-Ser, of rat IgA prevented poly-IgA formation (E. coli-produced proteins). **(B)**. Human recombinant (HEK293-produced proteins) poly-rIgA band disappeared with C471S mutation as shown by Western blotting using anti-human IgA alpha chain antibody. These results indicated that Cys471 residue of IgA heavy chain involved disulfide connections among self-aggregated rIgA units. R: reducing condition; NR: non-reducing condition.

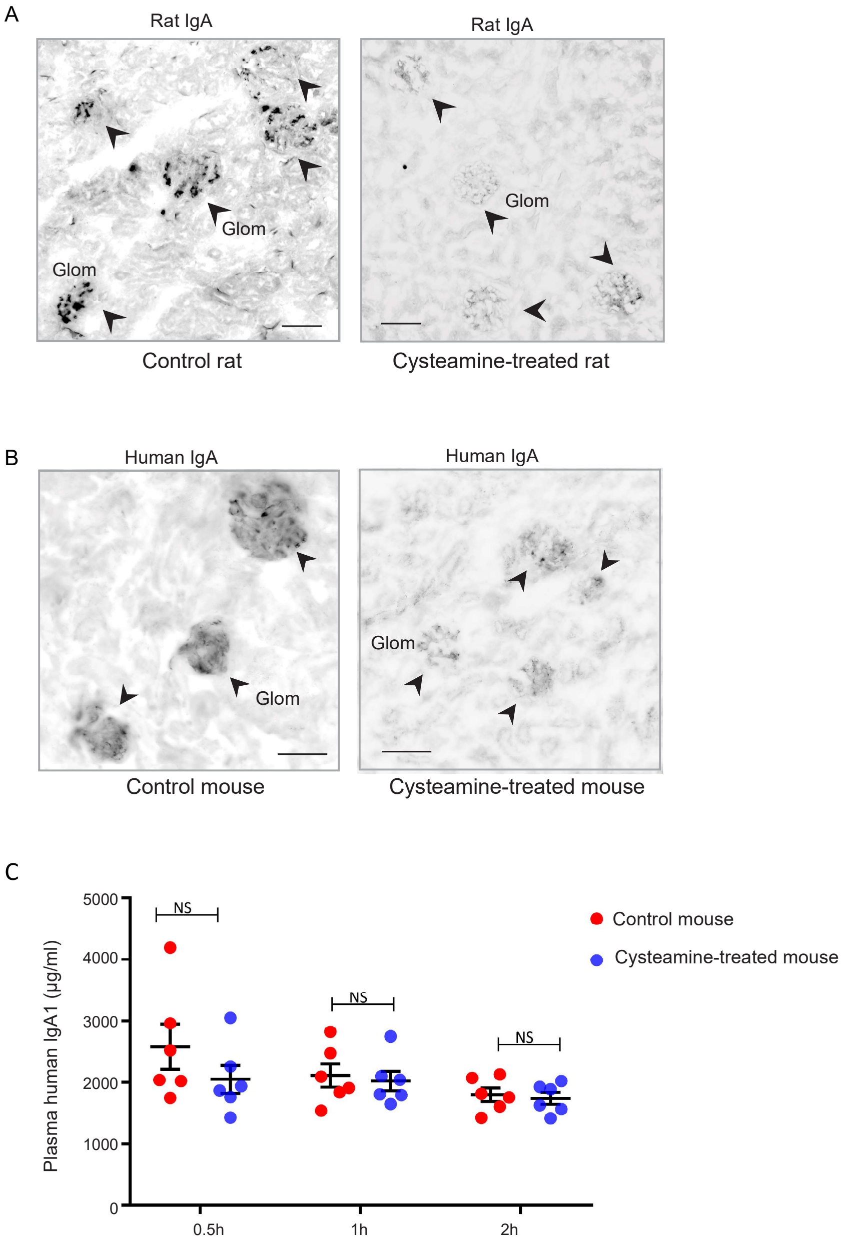

Supplementary Figure S4. In vivo treatment of rats and mice with cysteamine lowers IgA deposition in the kidney.

These immunofluorescence images were from the same study as in Figure 6, in which rats or mice incurred IgA deposition in the kidney following injections with either recombinant rat rIgA1 (to rats) or native human IgA extracted from human plasma (to mice). **(A)**. Prominent rIgA deposition in glomerular mesangium (arrowheads) was in control rats treated with buffer, in contrast to weaker IgA signals in glomeruli of cysteamine-treated rats. **(B)**. Prominent IgA1-deposition to the glomerular were found in control-treatment group of mice. Pretreatment of mice with cysteamine greatly reduced IgA1-deposits in the glomerulus. **(C)**. Plasma human IgA1 levels of mice at 0.5h, 1h and 2h after IgA1 injection did not show significant differences between control group and cysteamine-treated group. NS, not significant.

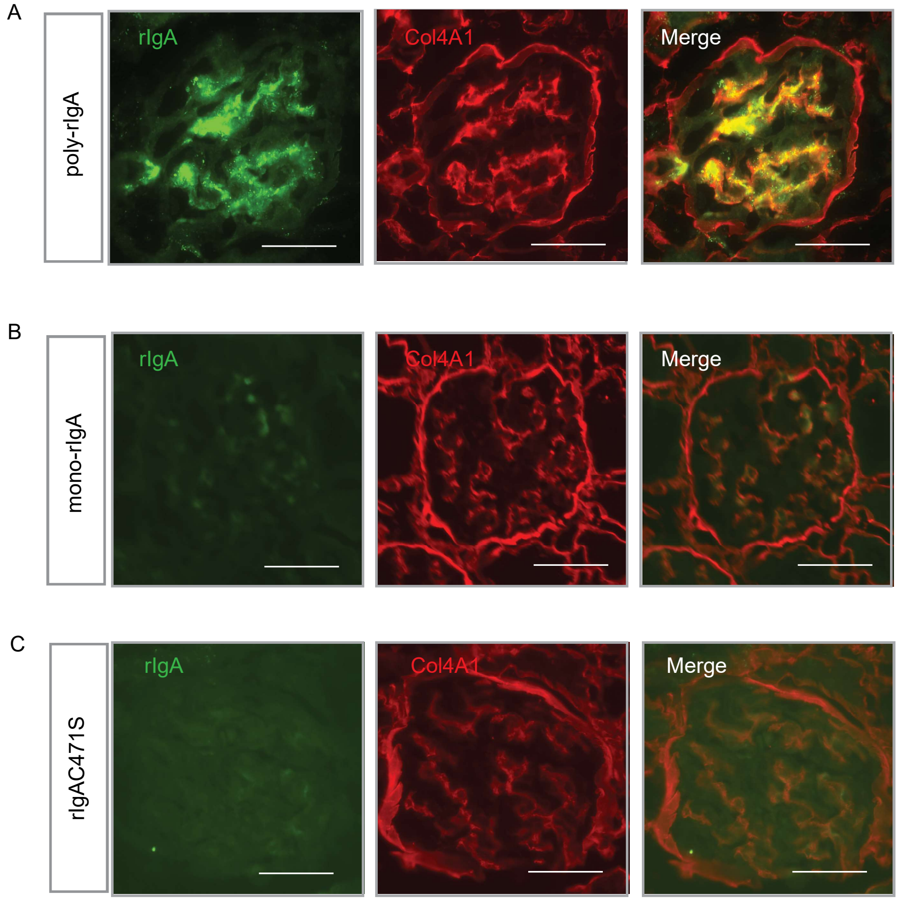

Supplementary Figure S5. Purified recombinant poly-IgA fraction causes glomerular deposition in rats.

Recombinant rat IgA was resolved by SEC. Poly-rIgA (~600 kDa) and mono-rIgA (~50 kDa) complexes were separately collected for i.v. injection studies, as well as recombinant rIgAC471S mutation. Rats (n=3 in each group) were injected with daily doses of either poly-IgA **(A)** or mono-rIgA **(B)** or rIgAC471S **(C)** for 5 consecutive days. Representative immunofluorescence staining images of the glomeruli are shown with IgA in green, Col4A1 in red. Scale bar 50µm.
